# Stereoscopic Imaging of Single Molecules at Plasma Membrane of Single Cell Using Photoreduction-Assisted Electrochemistry

**DOI:** 10.34133/research.0443

**Published:** 2024-08-13

**Authors:** Rong Jin, Yu Li, Yanyan Xu, Lei Cheng, Dechen Jiang

**Affiliations:** ^1^ State Key Laboratory of Analytical Chemistry for Life Science and School of Chemistry and Chemical Engineering, Nanjing University, Nanjing, China.; ^2^ College of Engineering and Technology, Southwest University, Chongqing, China.

## Abstract

Stereoscopic imaging of single molecules at the plasma membrane of single cell requires spatial resolutions in 3 dimensions (*x*-*y*-*z*) at 10-nm level, which is rarely achieved using most optical super-resolution microscopies. Here, electrochemical stereoscopic microscopy with a detection limit down to a single molecule is achieved using a photoreduction-assisted cycle inside a 20-nm gel electrolyte nanoball at the tip of a nanopipette. On the basis of the electrochemical oxidation of Ru(bpy)_3_^2+^ into Ru(bpy)_3_^3+^ followed by the reduction of Ru(bpy)_3_^3+^ into Ru(bpy)_3_^2+^ by photogenerated isopropanol radicals, a charge of 1.5 fC is obtained from the cycling electron transfers involving one Ru(bpy)_3_^2+/3+^ molecule. By using the nanopipette to scan the cellular membrane modified with Ru(bpy)_3_^2+^-tagged antibody, the morphology of the cell membrane and the distribution of carcinoembryonic antigen (CEA) on the membrane are electrochemically visualized with a spatial resolution of 14 nm. The resultant stereoscopic image reveals more CEA on membrane protrusions, providing direct evidence to support easy access of membrane CEA to intravenous antibodies. The breakthrough in single-molecule electrochemistry at the cellular level leads to the establishment of high-resolution 3-dimensional single-cell electrochemical microscopy, offering an alternative strategy to remedy the imperfection of stereoscopic visualization in optical microscopes.

## Introduction

The stereoscopic visualization of proteins in the plasma membrane of a single cell at the nanoscale is crucial for deeply understand their roles in complex cellular activities [1–3]. Owing to the recent breakthrough in single-molecule fluorescence detection, the emerging super-resolution optical microscopes have surpassed the optical diffraction limit and enabled the imaging of proteins with the spatial resolution down to 10 nm [4–6]. Despite the continuous improvements in super-resolution optical microscopy for single-cell imaging, the spatial resolution in the *Z* direction remains at 50-nm level, affecting the imaging quality in the stereoscopic observation [7–9]. Therefore, the development of stereoscopic imaging with high resolution in 3 dimensions (*X*-*Y*-*Z*) is still being actively pursued.

Multiple scanning probe microscopies have been developed to achieve a high-spatial stereoscopic image of the cell membrane. For example, atomic force microscopy (AFM) is the most popular tool in these microscopies [10,11]. However, the weak molecule interaction between the receptors at the AFM tip and target proteins at cellular membrane worsens the imaging resolution at ~100 nm [12]. Scanning electrochemical microscopy is another technique that could visualize molecules at the cellular membrane by collecting Faradic current from their electrochemical tags [13,14]. To improve the spatial resolution, scanning electrochemical cell microscopy (SECCM) was developed, utilizing a nanodroplet hung at the tip of a nanocapillary as an electrochemical cell [15,16]. Previously, our group has utilized SECCM to realize the stereoscopic imaging of membrane proteins with a spatial resolution of 160 nm [17]. The further development of electrochemical microscopies with the resolution down to a few nanometers and the sensitivity down to single molecules faces the difficulty of collecting sufficient charges from limited electrochemical probes. Only 1 to 2 electrons are transferred during one-molecule reaction, producing charges at the level of 10^−19^ C. This value is far below the recording limit (10^−14^ C/s) using a conventional electrochemical station. Accordingly, single-molecule electroanalysis is challenging and restricts the development of stereoscopic electrochemical imaging of proteins in single cells.

In 1995, Fan and Bard [18] first reported single-molecule electrochemistry by trapping a single electrochemical molecule between 2 electrodes. The molecule is electrochemically oxidized at one electrode and further electrochemically reduced at the other electrode. The extremely close distance between these 2 electrodes permits the continuous cycling of electrochemical reactions, enabling sufficient charge collection for the recording. To simplify single-molecule electrochemical detection, many novel electrode architectures, such as quadruple-barreled pipettes and self-aligned nanogaps, have been designed in recent years [19,20]. However, completing the redox-cycling amplification from electrochemical probes labeled on the cell remains challenging. Consequently, electrochemical imaging of cellular proteins at the single-molecule level has not yet been achieved.

**Fig. 1.**
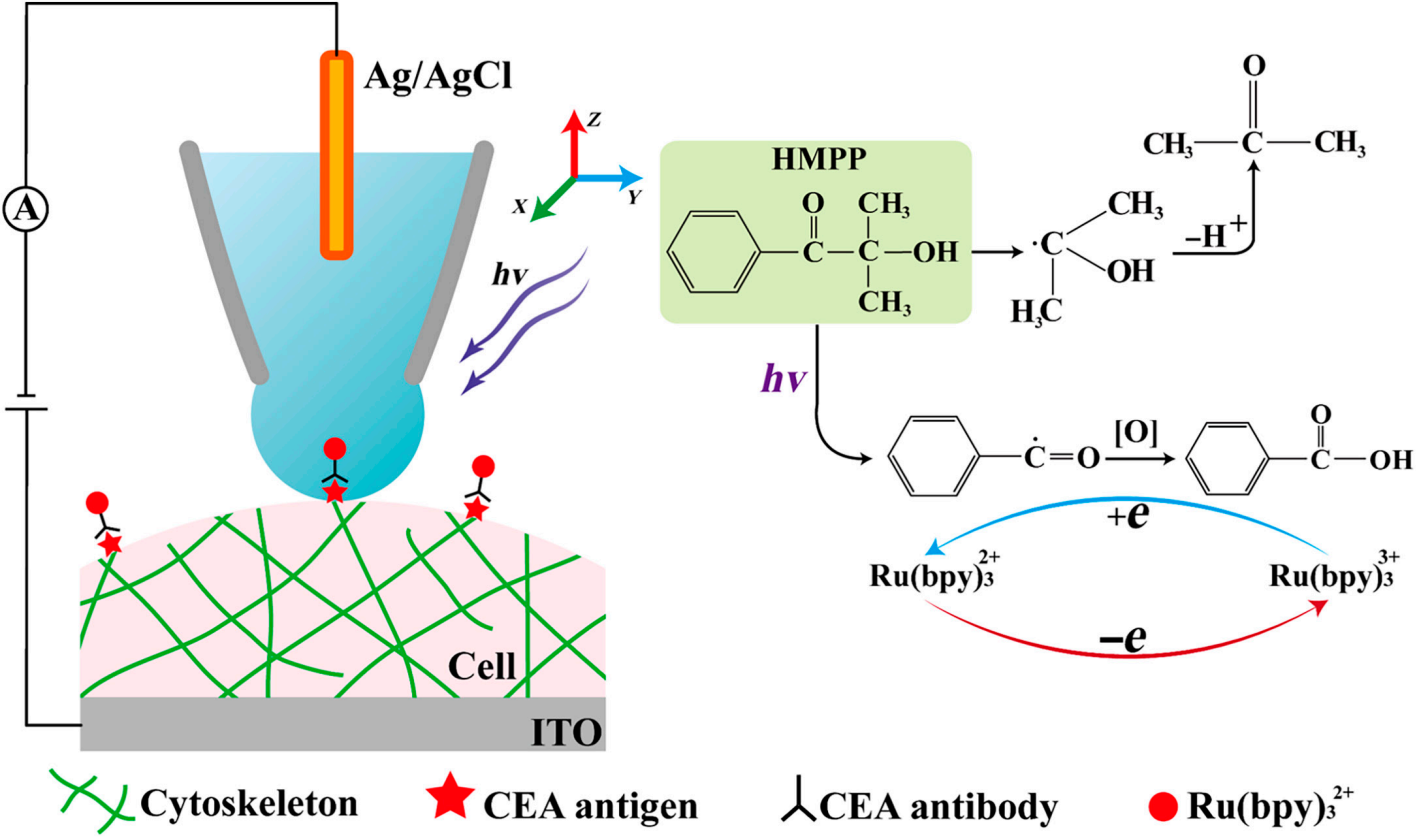
The schematic detection of single Ru(bpy)_3_^2+^-tagged immune complex at the cellular membrane using photoreduction-assisted electrochemical cycling. The right figure shows the detail reactions about the redox cycling, including the electrochemical oxidation and the subsequent photoreduction process.

Here, redox cycling, including the electrochemical oxidation of bis-(2,2′-bipyridine)-4′-methyl-4-carbox bipyridine-ruthenium N-succinimidyl ester-bis(hexafluorophosphate) [Ru(bpy)_3_^2+^] into Ru(bpy)_3_^3+^ followed by the reduction of Ru(bpy)_3_^3+^ into Ru(bpy)_3_^2+^ by photogenerated isopropanol radicals, is established inside a 20-nm gel electrolyte nanoball at the tip of a nanopipette. As illustrated in Fig. 1, a mixture, including acrylamide (AM), H_2_SO_4_, and the photoinitiator [2-hydroxy-2-methylpropiophenone (HMPP)], is filled into the nanocapillary as a gel electrolyte [21]. After polymerization under ultraviolet (UV) illumination, a 20-nm nanoball protrudes at the tip of the nanopipette. Compared with the previous method using the liquid droplet, the spatial resolution and stability are improved as the crystallization at the tip and the droplet collapsing are effectively restrained with the adoption of gel electrolyte [21]. Besides, the hydrophilicity limitation can be removed because of the relatively stable structure of the nanoball. Similar with typical SECCM principle [22–26], the 3-dimensional (3D) morphology is achieved by the feedback based on the contact of the nanoball with the cellular membrane. With the application of a positive potential at the supporting indium tin oxide (ITO) electrode, Ru(bpy)_3_^2+^ at the cell is electrochemically oxidized into Ru(bpy)_3_^3+^. As shown in Fig. 1, with continuous UV illumination, isopropanol radicals and benzaldehyde radicals will be generated by the remaining HMPP in the nanoball, where isopropanol radicals further turn into acetone [27–29]. Since benzaldehyde radicals are reported to be highly reductive [30], Ru(bpy)_3_^3+^ can be reduced into Ru(bpy)_3_^2+^, with the benzaldehyde radical itself being oxidized into benzoic acid. The generated Ru(bpy)_3_^2+^ participates in the subsequent electrochemical oxidation process and thus completes redox cycling to amplify the electrochemical current. Consequently, this new strategy enables the electrochemical measurement of membrane proteins at the single-molecule level, which promotes the development of high-resolution stereoscopic electrochemical microscopy for single-cell analysis.

## Results and Discussion

### Photoreduction-assisted redox cycling of Ru(bpy)_3_^2+/3+^

**Fig. 2.**
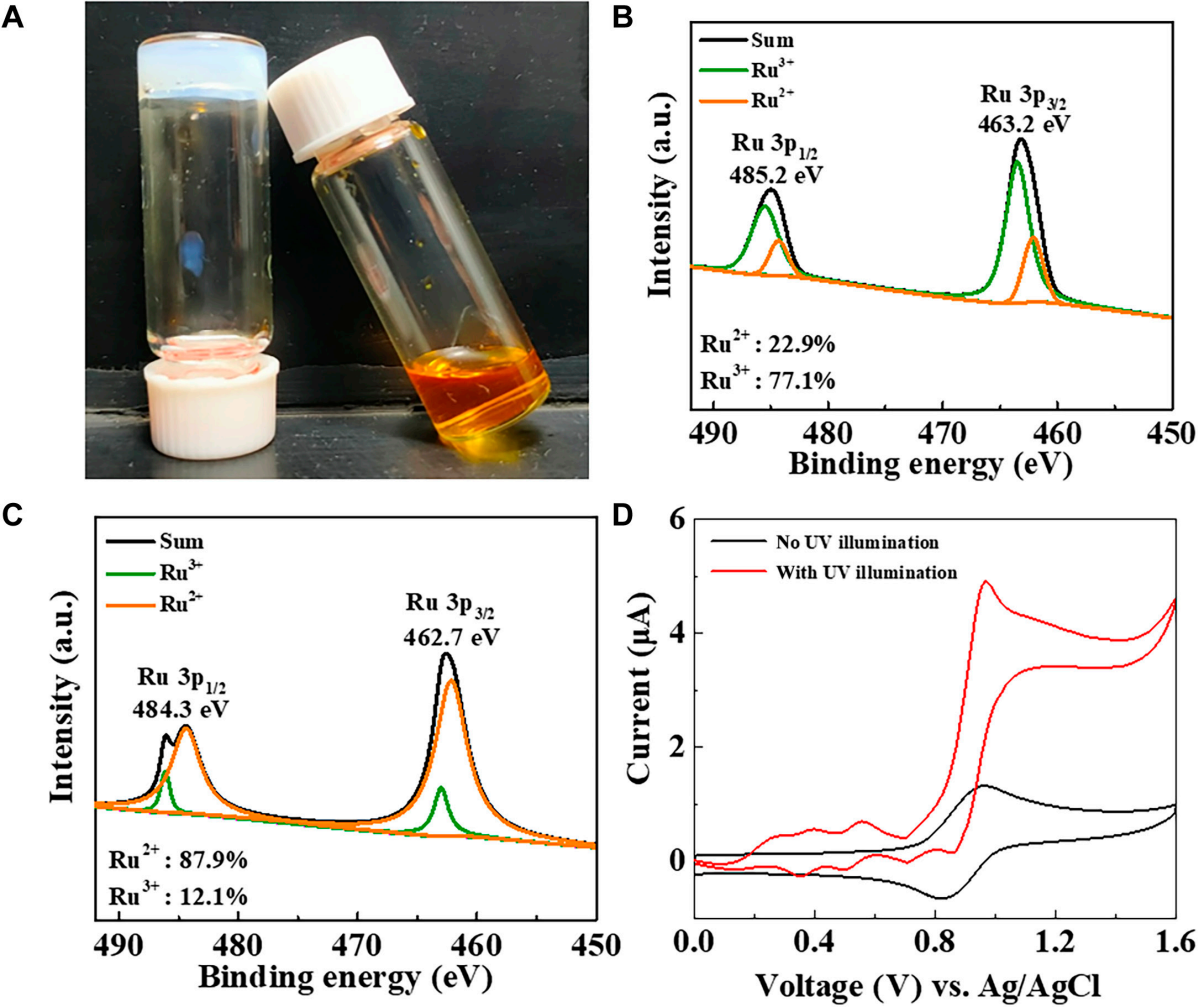
Electrochemical characteristics of Ru(bpy)_3_^3+^ in gel electrolyte. (A) The images about polymerized AM in presence of HMPP (left) and the aqueous state of the mixture containing AM, HMPP, and Ru(bpy)_3_^3+^ (right) after the exposure to the illumination for 5 min. (B) XPS analysis of HMPP solution with 77% Ru(bpy)_3_^3+^ and 23% Ru(bpy)_3_^2+^. (C) XPS analysis of HMPP solution with Ru(bpy)_3_^3+^ and Ru(bpy)_3_^2+^ after 5-min illumination. (D) The currents from 500 mM H_2_SO_4_ solution containing 0.1 mM Ru(bpy)_3_^2+^ and HMPP (1%, v/v) before and after the illumination. The scanning rate is 5 mV/s. a.u., arbitrary units.

To support the proposed mechanism of redox cycling, we performed the initial characterization in a solution containing HMPP. After exposure to UV illumination, mass spectroscopic analysis reveals the consumption of HMPP and the generation of benzoic acid in the solution (Fig. S1), which confirms the decomposition path of isopropanol and benzaldehyde radicals. Subsequently, a solution containing AM and HMPP was mixed with Ru(bpy)_3_^3+^ and exposed to illumination. Unlike the polymerization of AM in the absence of Ru(bpy)_3_^3+^, an organic solution persists in the presence of Ru(bpy)_3_^3+^ (Fig. 2A), illustrating the absence of polymerization. This result indicates that the presence of Ru(bpy)_3_^3+^ could remove the radicals so that the photopolymerization process is inhibited. X-ray photoelectron spectroscopy (XPS) analysis shows that after 5 min of illuminating the HMPP solution with 77% Ru(bpy)_3_^3+^ and 23% Ru(bpy)_3_^2+^, the contents of Ru(bpy)_3_^3+^ and Ru(bpy)_3_^2+^ changed to 12% and 88%, respectively (Fig. 2B and C). The decrease in the Ru(bpy)_3_^3+^ content and the associated increase in Ru(bpy)_3_^2+^ content provide evidence for the reduction of Ru(bpy)_3_^3+^ to Ru(bpy)_3_^2+^ by free radicals from HMPP.

The current through the redox cycling of Ru(bpy)_3_^2+/3+^ is investigated at ITO in a solution containing Ru(bpy)_3_^2+^ and HMPP. Before illumination, cyclic voltammetry records the reversible redox peaks associated with the electrochemical oxidation and reduction between Ru(bpy)_3_^2+^ and Ru(bpy)_3_^3+^ (Fig. 2D, black curve). The averaged peak current in the oxidation process from 3 independent measurements (*n* = 3) is 1.34 ± 0.17 μA. Under illumination, the oxidation current increases to 4.91 ± 0.51 μA (*n* = 3; Fig. 2D, red curve). The control experiment is conducted at ITO electrode in the absence of HMPP. The currents are close with and without the illumination (Fig. S3A), indicating the negligible effect on the electrochemical property of ITO from the illumination. Therefore, more oxidation from Ru(bpy)_3_^2+^ to Ru(bpy)_3_^3+^ in the presence of the photogenerated benzaldehyde radicals is verified. Meanwhile, the reduction current is nearly undetectable, suggesting the absence of electrochemical reduction of Ru(bpy)_3_^3+^ to Ru(bpy)_3_^2+^. With a gradually decreased scan rate, the voltammogram shifts from a duck shape to a sigmoidal shape, indicating the presence of an electrochemical reaction with reversible electron transfer, followed by irreversible chemical reaction (Fig. S2). These results support the occurrence of photoreduction of Ru(bpy)_3_^3+^ into Ru(bpy)_3_^2+^ as proposed, establishing the redox cycling of Ru(bpy)_3_^2+/3+^.

### Single-molecule SECCM imaging based on redox cycling of Ru(bpy)_3_^2+/3+^

To further investigate the redox cycling of Ru(bpy)_3_^2+/3+^ in the nanoball at the tip of the nanopipette, we filled AM, HMPP, and H_2_SO_4_ into the nanocapillary to prepare the gel electrolyte. H_2_SO_4_ is used to decrease the viscosity of the gel electrolyte in the pipette, thereby preventing adhesion between the nanoball and the scanning surface. The nanocapillary, characterized using scanning electron microscopy (SEM), exhibited an orifice of 18 ± 2 nm (*n* = 4) (Fig. S4A). After polymerization under illumination for 5 min, a nanoball protrudes at the tip of the nanopipette, serving as the electrochemical cell in SECCM (Fig. S4B). According to our previously developed protocol [21], the nanoball is in contact with an Au-coated ITO electrode, and a voltage of 1.4 V is applied at the Au/ITO electrode to initiate corrosion. The SEM image (Fig. S5) illustrates that the corrosive hole at the electrode has an average diameter of 14 ± 2 nm (*n* = 36), which represents the contact region between the nanoball and the scanning surface. No crystal is observed at the contact region, exhibiting that the electrolyte is locked into the cross-linked structure of hydrogel electrolyte. To characterize the stability of nanoball under the illumination, we performed the continuous cyclic voltammetry (CV) measurements during the contact of ITO electrode. The similar current traces are observed (Fig. S3B), which illustrates the stable nanoball even after the additional illumination.

**Fig. 3.**
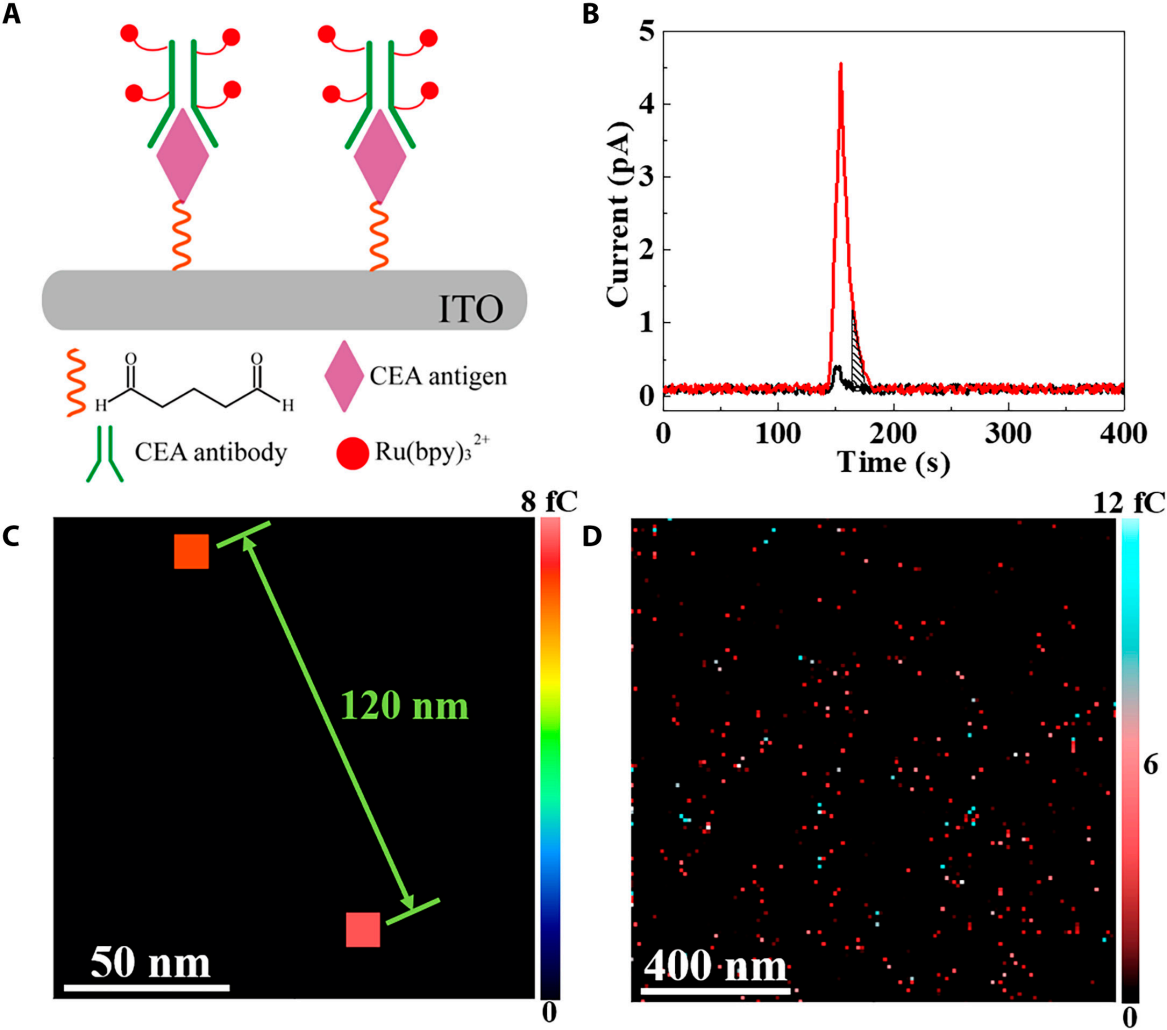
Single-molecule imaging results using SECCM. (A) The schematic structure of Ru(bpy)_3_^2+^-tagged antibody–antigen immune complex at glutaraldehyde-modified ITO surface. (B) The SECCM contact current with (red curve) and without (black curve) UV illumination. (C) The SECCM image of 2 antigen–antibody complexes colabeled by Cy5 and Ru(bpy)_3_^2+^. (D) The enlarged SECCM image of individual immune complexes on the ITO surface. The scanning interval is 10 nm.

To demonstrate the detection ability at the single protein level, we bound Ru(bpy)_3_^2+^-tagged antibodies with the carcinoembryonic antigen (CEA) antigen on the glutaraldehyde-modified ITO surface, forming a monolayer of immune complexes. The structure of this complex is illustrated in Fig. 3A. Following our established protocol, the density of CEA on the surface could be adjusted to illustrate the single-molecule distribution [31]. To validate the single-molecule distribution, we colabeled 2 Cy5 molecules with one molecule of Ru(bpy)_3_^2+^-tagged immune complex, enabling simultaneous fluorescence observation (Fig. S6A). Fluorescence imaging using stimulated emission depletion (STED) microscopy displays a clear 2-step photobleaching at single immune complex, corresponding to 2 molecules of Cy5 (Fig. S6B to D). The fluorescence result supports the formation of single molecule of complex at the surface. Then, 2 complexes with a distance of 120 nm are selected for the following electrochemical imaging. Upon contact of the nanoball with these 2 molecules of immune complexes, chronoamperometry (CA) was used to measure the current from the redox cycling of Ru(bpy)_3_^2+/3+^. A voltage of 1.23 V, sufficient to electrochemically oxidize Ru(bpy)_3_^2+^, was applied to the ITO surface. When the nanoball contacted the ITO region with the immune complexes, the initial pulse current was primarily a non-Faradic current due to the formation of the circuit between the Ag/AgCl electrode inside the nanopipette and the supporting ITO slide (Fig. 3B, black trace). Given that the resistance capacitance (RC) constant (*τ*) is determined to be 6.6 ± 0.87 ms (*n* = 3; Fig. S7 and Table S1), a non-Faradic current should not be present after 20 ms (3*τ*). Experimentally, no current was observed after 20 ms, indicating the complete decay of the non-Faradic current. No Faradic current was collected in the following 10 ms, demonstrating the infeasibility of detection of Ru(bpy)_3_^2+^ without redox cycling.

When the illumination was activated, an obvious increase in the current was observed (Fig. 3B, red trace). Notably, the observation of the Faradic current from 20 to 30 ms exhibited the occurrence of redox cycling of Ru(bpy)_3_^2+/3+^ assisted by photoreduction. The charges during this period (shadow region in Fig. 3B) were integrated to be 6 ± 1 fC. This charge increase corresponds to 4 Ru(bpy)_3_^2+^ tags [1.5 fC per Ru(bpy)_3_^2+^ molecule] at one immune complex, amounting to approximately 9,500 cycles between Ru(bpy)_3_^2+^ and Ru(bpy)_3_^3+^. The time for one cycle is calculated to be 1.0 μs. Considering that the time for the electron transfer from Ru(bpy)_3_^2+^ to Ru(bpy)_3_^3+^ in the gel electrolyte is 0.64 ± 0.04 μs (*n* = 3; Fig. S8 and more description in the Supplementary Materials), the completion of one cycle, including electrochemical oxidation and subsequent photoreduction, in 1.0 μs is reasonable, given that the electrochemical noise is determined to be 0.15 pA (Fig. S9). Therefore, the signal can be distinguished easily as it is always larger than 1 pA. The SECCM charge image displays that the 2 pixels with increased charge have the same distance of 120 nm (Fig. 3C), compared to Fig. S6B and C. The consistency in the fluorescence and SECCM images confirms the observation of a single molecule of the immune complex using SECCM.

After the confirmation of single-molecule imaging, the entire surface modified with the immune complexes was scanned. Among all the spots with the increased Faradic charges, ~70% of single spots exhibit charges of 6 ± 1 fC (Fig. 3D). Most of the spots occupy one pixel (14 nm), indicating only one complex in each spot. It is noted that ~30% of single spots show charges of 12 ± 2 fC, likely due to the aggregation of the 2 complexes. Considering that the gyration radius of the CEA antigen is approximately 8 nm [32], the existence of aggregation in one pixel is highly possible. Increasing the amount of CEA at the electrode surface could introduce more antibodies and the resulting Ru(bpy)_3_^2+^-tagged complexes. The SECCM image (Fig. S10) displays the continuous distribution of the complex monolayer on the surface, including more aggregations of the antigens. The observation of more antigens on the surface further supports that our electrochemical imaging could accurately reflect the distribution of antigens on the surface.

### Single-molecule stereoscopic visualization of membrane proteins in single cells

Following the validation of SECCM visualization at the single-molecule level, MCF-7 cells with up-regulated CEA on the plasma membrane were used as the cell model. To facilitate the immune binding of the antibody to the membrane antigen on the cell surface, we applied the fixation process. The process, widely used in molecular cytogenetic and cancer research, does not alter the distribution of CEA in the membrane [33]. Moreover, the fixation and subsequent dehydration could increase membrane permeability for a low resistance that favors the SECCM measurement. After the immune binding of Ru(bpy)_3_^2+^-tagged antibodies with CEA on the cell surface, the cellular membrane is scanned by the nanoball to obtain the morphology and current measurements before and after illumination. Since the electrochemical reaction of SECCM is restricted inside the nanodroplet, the spatial resolution of this technology is strictly decided by the diameters of the hanging droplet at the tip. Considering the diameter of the droplet is almost the same as the size of the nanocapillary tip, single cell is scanned using nanopipettes with tip diameters of 20 and 80 nm. The images are shown in Fig. S11. As labeled by the red and green arrows in Fig. S11B, tiny pseudopodia can only be observed with 20-nm nanopipette. This result supports the conclusion that a high resolution can be achieved using a small size of nanopipette and more details of the cell surface can be observed.

**Fig. 4.**
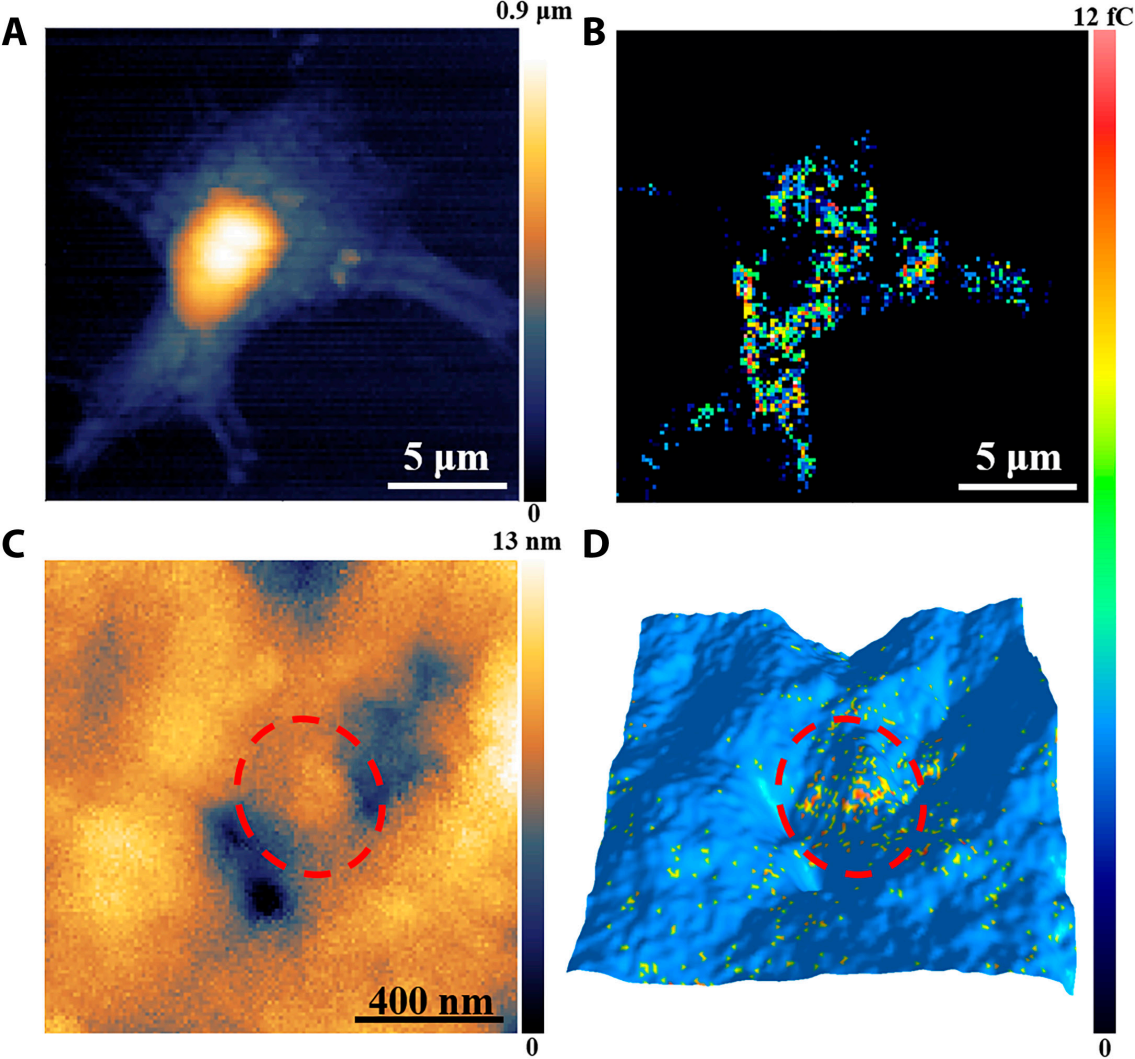
Antigen imaging results at MCF-7 plasma membrane using SECCM. (A) The SECCM topography image of single MCF-7 cell. (B) The SECCM charge image to show the distribution of CEA antigen at the cellular membrane. The scanning region in (A) and (B) is 20 μm × 20 μm (128 pixels × 128 pixels), and the scanning interval is 156 nm. (C) The SECCM 3D image to show the region with cellular protrusions and caveolae. (D) The overlapping SECCM charge and 3D topography image to stereoscopically illustrate the distribution of CEA. The scanning region in (C) and (D) is 1.28 μm × 1.28 μm (128 pixels × 128 pixels), and the scanning interval is 10 nm. The charge is integrated from the currents recorded from 20 to 30 ms.

The SECCM topography (Fig. 4A) displays a high region (~800 nm) at the cell nucleus, a low cellular region (less than 400 nm) surrounding the nucleus, and pseudopodia characteristics. Subsequently, the currents from the low cellular region are analyzed. The CA measurement under 1.23 V gives a larger Faradic current with illumination than without illumination (Fig. S12), signifying the occurrence of redox cycling from Ru(bpy)_3_^2+^ at the cellular membrane. The conductivity of the cellular membrane after the fixation and dehydration is due to the presence of cytoskeleton, as observed in various cells (breast cells, hippocampal neurons, and PC12 cells) using conductive AFM [34,35]. Following their protocol, a disk nanoelectrode with a diameter of 180 nm was used to contact the cellular membrane modified with Ru(bpy)_3_^2+^ to perform cyclic voltammetry. The voltammogram exhibits the oxidation and reduction peaks at 1.37 and 0.71 V, respectively (Fig. S13). In contrast, no oxidation or reduction peak is observed when the membrane is not linked with Ru(bpy)_3_^2+^. All these findings support that the membrane conductivity leads to the collection of redox current at the cellular membrane. The ionic resistance and time constant are measured to be (7.25 ± 0.87) × 10^12^ Ω and 5.9 ± 0.52 ms (*n* = 5), respectively (Fig. S14), which are similar to the values from the measurement of the immune complex-modified surface [(5.49 ± 0.63) × 10^12^ Ω and 4.3 ± 0.61 ms; *n* =5; Table S1].

After scanning the entire cell under illumination, the SECCM charge image illustrates that the Faradic charges at each contact region range between 0 and 12 fC (Fig. 4B). Referring to the charges collected from the single Ru(bpy)_3_^2+^-tagged antibody–antigen complex (6 fC), the observation of Faradic charges between 0 and 12 fC indicates 1 or 2 molecules of CEA at one contact region. Once the illumination is turned off, the Faradic charges disappear because of the cessation of redox cycling. To confirm the preference of Ru(bpy)_3_^2+^-tagged antibody–antigen complexes at the cellular membrane, we introduced Cy5- and Ru(bpy)_3_^2+^-cotagged antibodies to bind the membrane antigen. The fine observation of Cy5 using STED fluorescence microscopy and Ru(bpy)_3_^2+^ using SECCM is applied at the same cell margin. The images show that all the contact regions with Faradic charge are located at the cellular margin, confirming the observation of Ru(bpy)_3_^2+^-tagged immune complex at the cell membrane (Fig. S15). To exclude the possibility of nonspecific binding of Ru(bpy)_3_^2+^-tagged antibodies to the cell membrane, we used J774 cells, which do not express CEA, as the control group. After culturing J774 cells in medium with Ru(bpy)_3_^2+^-tagged antibodies, almost no Faradic charges are observed in the SECCM image, suggesting no nonspecific adsorption of Ru(bpy)_3_^2+^-tagged antibodies on the cellular membrane in the absence of CEA antigen (Fig. S16). All these results confirm that the Faradic charge observed in MCF-7 cells under illumination is associated with the photoreduction-assisted redox process of the Ru(bpy)_3_^2+^-tagged immune complex at the cellular membrane.

Considering the cell is nonviable after fixation and dehydration, the cell membrane should not change during the long scanning process. Therefore, replicate scanning of the cell membrane should yield consistent information to validate the imaging accuracy. Experimentally, the replicate scanning of the whole cell exhibits nearly identical morphological and charge images (Fig. S17), illustrating good reproducibility in single-cell SECCM imaging. To extensively investigate the imaging error during scanning, we conducted fine imaging (128 pixels × 128 pixels, 10-nm scanning interval) of a small cell membrane region (1.28 μm × 1.28 μm) (Fig. S18). Differences between 2 consecutive scans shows that only 97 and 142 of 16,384 points (0.59% and 0.87%) are mismatched in the morphological and charge images, respectively. These minimal mismatches confirm the high reproducibility of SECCM imaging. The total Faradic charges at the entire cellular surface are calculated to be 420 ± 88 pC (70,000 ± 15,000 molecules, *n* = 3), corresponding to 23 ± 4.8 fg of CEA at one MCF-7 cell. This value is consistent with previous results obtained using other methods (18 to 27 fg), supporting the accuracy of our SECCM measurement [36]. To our knowledge, this is the first report to illustrate single-cell electrochemical imaging at the single-molecule level.

The detailed analysis of the charges in the SECCM image exhibits an uneven distribution of CEA at the cellular membrane. Fewer antigens are recorded at the nucleus region, which might be ascribed to high resistance in this region, resulting in a smaller current. Consequently, these values are excluded from the analysis as they do not accurately reflect the real distribution of CEA. In the surrounding cellular regions, more CEAs are observed at the cell periphery, particularly in areas with long pseudopodia that direct cell migration. Many studies have revealed that CEAs are intended to interact with integrins at the membrane, which are connected to the actomyosin cytoskeleton to drive cell migration [37–40]. Thus, the observation of a rich CEA region toward the migration direction provides direct evidence to elucidate the molecular mechanism of directional cell migration. As a cell-surface-bound glycoprotein, CEA plays an important role in activating cells, such as cell adhesion, spreading, proliferation, and migration. This CEA-mediated signaling involves the clustering of CEA at the membrane microdomains via the activation of integrin α_5_β_1_ and the associated specific elements [41]. Since integrin is connected to the actin cytoskeleton, the microdomains with CEA clusters should be mainly distributed at the membrane protrusion. This feature allows for easy access of membrane CEA to intravenous antibodies, which makes CEA an excellent target for antibody-based therapy.

To deeply study the distribution of CEA at the microdomain, we chose a small membrane region including cellular protrusions and caveolae for scanning. The SECCM topography image shows the structure of the protrusions and cavitations at the membrane with a height difference of 13 nm (Fig. 4C). Simultaneously, the overlapping image of the charge and the 3D topography displays a significantly high level of CEA at the protrusions (Fig. 4D) compared with those at the caveolae and the surrounding regions (inside the red circle). The observation of more CEA clusters in this region clearly demonstrates the assembly of CEA in integrin-rich areas to form an action cytoskeleton, presenting the accuracy of high-resolution electrochemical imaging. Overall, the established high-resolution single-cell electrochemical imaging not only addresses the long-lasting challenge in electrochemistry for the visualization of single molecules in the cell but also provides detailed molecular information to facilitate the understanding of their cellular roles.

In summary, a high-resolution stereoscopic image of proteins in a single cell is achieved by establishing photoreduction-assisted single-molecule electrochemistry. The designed photoreduction of Ru(bpy)_3_^3+^ to Ru(bpy)_3_^2+^ completes the redox cycling between Ru(bpy)_3_^2+/3+^, amplifying the electrochemical signal and recording 1.5 fC from a single-molecule reaction for electrochemical visualization. The new strategy eliminates the need for the traditional 2 close-electrode setup for single-molecule electrochemistry, making it suitable for single-cell imaging with a spatial resolution down to 14 nm. The observation of more CEA clusters at cellular protrusions provides evidence for the role of CEA in antibody-based therapy. Currently, more proteins are being visualized in the laboratory using the corresponding Ru(bpy)_3_^2+^-tagged antibodies, further establishing high-resolution electrochemical microscopy for biological studies.

## Materials and Methods

### Preparation of gel electrolyte inside nanopipettes

The nanocapillary (BF100-58-10, Sutter Instrument, CA, USA) was prepared using a P-2000 micropipette puller (Sutter Instrument) to produce a ~20-nm opening. The preparation of gel nanopipettes followed a previously reported protocol [*18*]. Briefly, 500 mM H_2_SO_4_ solution containing 50 wt % of AM, 20 wt % of hydroxyethyl acrylate, and 30 wt % of polyethylene glycol diacrylate was injected into the nanocapillary. The ratio of water and monomer was 6:4. The photoinitiator used was HMPP with a content of 1 vol %. The light source was 365-nm UV light with a power of 6 W. Polymerization was conducted under UV light for 5 min to obtain the gel electrolyte. The morphology of the nanopipette was characterized by SEM (Hitachi S-4800, Japan).

### Synthesis of Ru(bpy)_3_^2+^-tagged CEA antibody.

Biotinylated CEA antibody [mouse anti-CEA (B5)/biotin] was obtained from Bioss Biotechnology Co. Ltd. (Beijing, China). Ru(bpy)_3_^2+^ and streptavidin were purchased from Sigma-Aldrich. Briefly, Ru(bpy)_3_^2+^ (1 mg/ml) and streptavidin (0.1 mg/ml) were reacted with stirring for 2 h at 4°С. The mixed solution was purified by ultrafiltration using a 10,000-molecular-weight cutoff membrane (Millipore, USA). The concentrated streptavidin-modified Ru(bpy)_3_^2+^ complex was diluted to 20 μg/ml with phosphate-buffered saline (pH 7.4) and stored at 4°C. To link the Ru(bpy)_3_^2+^ complexes to the antibodies, the streptavidin-associated Ru(bpy)_3_^2+^ complexes were incubated with the biotinylated CEA antibodies at 37°С for 30 min.

### Coupling of Ru(bpy)_3_^2+^- and/or Cy5-tagged CEA antibody with the antigens

ITO electrodes (SPI Supplies, USA) were cut into pieces of 10 mm × 20 mm and cleaned by sonication in acetone, ethanol, and water for 10 min each. Then, they were dipped into a solution of 30% H_2_O_2_, NH_3_·H_2_O, and H_2_O in a 1:1:5 (vol) for 30 min and washed 3 times with water. Finally, the ITO electrode was silanized with 1% 3-aminopropyltriethoxysilane in ethanol at 80°C for 30 min. After washing 3 times with water to remove the physically absorbed silanes, the electrodes were immersed in 5% glutaraldehyde at room temperature for 60 min. The electrodes were then immersed in 25 pM CEA antigen for 60 min at 37°C, followed by antibody (biotin-CEA antibody; 500 ng/ml) for 60 min at 37°C. Then, the electrodes were washed with water and immersed in 50 pM streptavidin-Ru(bpy)_3_Cl_2_ for 60 min at 37°C. For the fluorescence imaging, the electrodes were immersed in 50 pM streptavidin-Cy5 for 60 min at 37°C before the modification with Ru(bpy)_3_^2+^. Considering that there are several biotins on the biotinylated antibody, this modification process should introduce both of Ru(bpy)_3_^2+^ and Cy5 at the immune complex. Finally, the electrodes were washed and dried at room temperature. Copper wire was used to connect the electrodes for subsequent electrochemical and single-molecule fluorescence tests.

To achieve the modification of Ru(bpy)_3_^2+^-tagged CEA antibody with the antigens at the cellular membrane, MCF-7 cells, and J774 cells were cultured on ITO electrodes at 37°C. Cells were fixed with 4% paraformaldehyde in the dark for 30 min. After dehydration, cells were exposed to 5 μM Ru(bpy)_3_^2+^-CEA antibodies for 30 min, during which Ru(bpy)_3_^2+^-CEA antibodies were linked with CEA at the cellular membrane.

### SECCM imaging

The SECCM apparatus used in our experiments was a home-built setup, which included the scanning, location, and control modules. More detailed description about the SECCM setup is shown in the Supplementary Materials. The ITO electrode with the immune complex layer or the cells was installed on the sample stage, which was connected with the data acquisition card via a copper wire. An Ag/AgCl wire was inserted into the nanopipette and connected with the data acquisition card through the copper wire. The voltage applied to the ITO electrode was 1.23 V. The current threshold was set at 0.04 pA. The gel nanopipette, installed on the *Z*-direction piezo, was initially positioned 200 μm away from the ITO electrode. During the approach of the nanopipette toward the sample, a combination of crude adjustment by *Z*-direction motor and fine adjustment by Z-direction piezo (travel range, 30 μm; step, 1 nm) was used to control the position of the nanopipette. Once the current threshold was detected, the position was recorded for the morphology analysis. In the following scanning process, the hopping mode was adopted, with the hopping height referenced to the initially recorded position. Because of the tiny overshoot of *Z*-direction piezo, the scanning speed of *Z*-direction piezo was restricted to 10 μm/s, resulting in an overshoot of approximately 2 nm. The footprint of the individual contact regions was characterized by SEM.

During the contact period (30 ms) of the gel nanoball at the tip of the nanopipette with the sample, currents were recorded. The currents during the last 20 ms of contact were integrated to calculate the amount of Ru(bpy)_3_^2+^, which could be used to quantify the antigens. Data analysis was performed using MATLAB 2018a. The scanning time depended on the contact points (array density) at the sample controlled by the region size and the scanning interval. Since a gel nanopipette was used, the sample could be scanned repeatedly. Therefore, the adaptive scanning algorithm was adopted to expedite high-spatial imaging. The detailed description about the adaptive scanning algorithm and the resultant scanning time is provided in the supporting information. For a typical image with an array density of 64 × 64 (region size, 640 nm × 640 nm; the interval, 10 nm), the imaging time was 22 min.

### Coimaging using SECCM and STED fluorescence microscopies

The immune complex or cells cotagged with Cy5 and Ru(bpy)_3_^2+^ were prepared on the ITO electrode. A selected area was imaged by STED (Leica SP8 STED 3X, Germany), including an inverted DMI6000 CS microscope equipped with a tunable (470 to 670 nm) pulsed white light laser (repetition rate of 78 MHz) for excitation and an STED laser for depletion. For the STED imaging, a HyD detector and 100× oil immersion objective (numerical aperture, 1.4) were used. The imaging parameters were set as follows: a format of 8,192 × 8,192, a speed of 100 Hz, a line average of 3, a frame average of 3, and a smart gain of 150%. The dyes were excited with the Cy5 tunnel chosen by the software. The recorded images were first processed using the Huygens Deconvolution software (Scientific Volume Imaging) and further processed with the ImageJ software. For the observation of fluorescence blinking from single molecules, a continuous shot of 650 nm with the interval of 50 ms was applied. The point spread function of individual molecules was localized by the 2D Gaussian function. To localize the centroid position of single immune complex in each frame with a high precision, the results were fitted with a Gaussian function for computational convenience.

After obtaining a high-spatial fluorescence image, the ITO electrode was mounted on the sample stage of SECCM. The nanopipette was positioned above the same selected area. The scanning pixels were set to 128 × 128 with the interval of 10 nm to achieve the highest spatial resolution. The hopping height was set to be 0.2 μm.

## Data Availability

All data related to the results and discussion are present in this paper and the Supplementary Materials.
