# Supplementary material for: Stereoscopic Imaging of Single Molecules at Plasma Membrane of Single Cell Using Photoreduction-Assisted Electrochemistry
Source: Research (Wash D C). 2024 Aug 13;7:0443. doi: 10.34133/research.0443 (PMC11319615; doi:10.34133/research.0443)
Supplement: Supplementary 1 — Figs. S1 to S18 Table S1 [file research.0443.f1.docx]

Supporting Information for

Stereoscopic Imaging of Single Molecules at Plasma membrane of Single Cell using Photoreduction-assisted Electrochemistry

Rong Jin^1^, Yu Li^1^, Yanyan Xu^1^, Lei Cheng^2^, Dechen Jiang^1*^

Rong Jin, Yu Li, Yanyan Xu, Dechen Jiang^*^

State Key Laboratory of Analytical Chemistry for Life and School of Chemistry and Chemical Engineering, Nanjing University, China

Address correspondence to: dechenjiang@nju.edu.cn

Lei Cheng

College of Engineering and Technology, Southwest University, China

Table of Content

Detail about the SECCM setup.

Detail about the scanning resolution and time.

Fig. S1. The ESI-MS spectroscopy of HMPP before and after illumination.

Fig. S2. CV results of 0.1 mM Ru(bpy)_3_^2+^ and HMPP (1% V/V) under illumination.

Fig. S3. CV results of ITO electrode and nanopipette filled with hydrogel with and without UV illumination.

Fig. S4. SEM images of the nano-capillary.

Fig. S5. SEM image of the footprints.

Fig. S6. STED result of single molecules.

Fig. S7. The initial non-Faradic current upon the contact.

Fig. S8. CV results in solid electrolyte with different scan rates.

Fig. S9. The typical electrochemical noise in the SECCM setup.

Fig. S10. The SECCM charge image of immune-complexes at ITO electrode.

Fig. S11. SECCM morphology of the same single cell using nanopipette with different tip diameters.

Fig. S12. The current traces of the nanoball contacted with Ru(bpy)_3_^2+^ tagged cell membrane at ITO electrode.

Fig. S13. The initial non-Faradic current upon the contact.

Fig. S14. CV results of cell surface using SECCM with scanning rate of 5 V/s.

Fig. S15. The STED fluorescence and SECCM images of the cell margin tagged with Ru(bpy)_3_^2+^ and Cy5.

Fig. S16. SECCM result of single J774 cell.

Fig. S17. The images from the replicate scanning of the whole cell tagged with Ru(bpy)_3_^2+^.

Fig. S18. The images from the replicate scanning of a small cell region.

Table S1. The stimulated result from the initial part of non-Faradic current.

**Detail about the SECCM setup.**

The home-built SECCM was divided into the scanning, location and control modules (the detail description about the setup was shown in supporting information). The scanning module was combined by an XY-direction piezo actuator (P-621, PI Co., Germany) and a Z-direction piezo actuator (P-753, PI Co., Germany). Both piezo actuators were driven by a servo controller (E-509, PI Co., Germany). The movement in Z direction was separated from XY direction to decrease the location error. The sample stage was installed on XY-direction piezo and the nanopipette was installed on Z-direction piezo. The current was amplified by a trans-impedance amplifier (DLPCA-200, FEMTO, Germany). The location module was combined by an XY-direction stepping motor and a Z-direction stepping motor (M-111.1, PI Co., Germany). Both motors were controlled by a motor controller (C-863.11). The XY and Z-direction piezo were installed on XY and Z-direction motors, respectively. The scanning module and the location module was shielded inside a Faraday cage, which was installed on an optical microscope (Ti, Nikon, Japan). The control module was combined by two home-made data acquisition cards with the sample frequency of 10 kHz. One card was used to apply voltage at the electrochemical cell and collect current. The other one was used to output analog voltage to the servo controller and the motor controller for the collection of the feedback signals. The communication between the control module and PC was constructed by a cable. The scanning program was written in C++ language.

**Detail about the scanning resolution and time.**

The scanning time depended on the contact points (array density) at the sample, which were controlled by the region size and the scanning interval. Since all-solid nanopipette was used, the sample could be scanned repeatedly. Therefore, the adaptive scanning algorithm was adopted to speed up high-spatial imaging. Typically, the sample was pre-scanned with a hopping mode of 5 μm. The pixel in XY-direction was set to be 32 × 32. For each scanning point, the times for the approaching, detection and withdrawn were approx. 500 (depending on the sample height), 30 and 50 ms, respectively. Then, the sample was moved to next point and wait for 200 ms to achieve a stable current base line. The whole time was about 780 ms for each scanning point and 13 min for this crude pre-scanning.

In the following fine scanning, one pixel was divided into 4 pixels. They shared the same recorded Z-direction position so that the hopping height could be adjusted to be 1 μm. Therein, the approaching and withdrawn times decreased to 100 and 10 ms, respectively. The time for each scanning point was about 330 ms. The imaging time with an array of 64 × 64 was 22 min.

For the super-fine image with an array of 128 × 128, the pixel was further divided into 4 pixels and the hopping height was shortened into 0.5 μm. By sharing the previously recorded Z-direction positon, the time for one pixel was further shortened at about 285 ms. The whole imaging needed 68 min.

**Fig. S1.** The ESI-MS spectroscopy of HMPP (5% V/V) before (A) and after (B) the illumination for 5 min.

**Fig. S2.** The cyclic voltammetry results of 0.1 mM Ru(bpy)_3_^2+^ and HMPP (1% V/V) under illumination with different scan rates (2, 5 and 20 mV/s).

Note: The process in our study can be considered as a typical EC’ reaction (an electrochemical reaction coupled by a chemical reaction). When the scanning speed of CV is low enough, i-E curve loses its peaks. The current can be described as:

$i=\frac{nFAC_{O}^{*}\sqrt{Dk^{'}C_{Z}^{*}}}{1+exp\left[ \frac{nF}{RT}\left( E-E_{1/2} \right) \right]}$ eq. S1

where *A* is the electrode area, *D* is the diffusion coefficient of the benzaldehyde radical, *k*’ is the standard reaction rate constant, *C*_Z_^*^ is the concentration of the benzaldehyde radical, *E*_1/2_ is the half-wave potential. As the chemical reaction can be usually regarded as a first order reaction, *k*’*C*_Z_^*^ can be described as *nF*/*RT*, eq. S1 can be simplified to be:

$i=\frac{FAC_{O}^{*}\sqrt{D\frac{F}{RT}}}{1+exp\left[ \frac{F}{RT}\left( E-E_{1/2} \right) \right]}$ eq. S2

According to the black line in Fig. S2, the current at half-wave potential is about 3.166 μA. *D* is calculated to be about 1.12×10^-5^ cm^2^·s^-1^.

**Fig. S3.** (A) CV results of ITO electrode in Ru(bpy)_3_Cl_2_ solution with (red curve) and without (black curve) UV illumination; (B) CV results of the nanoball at the tip of nanopipette in contact with ITO electrode with (red curve) and without (black curve) UV illumination.

**
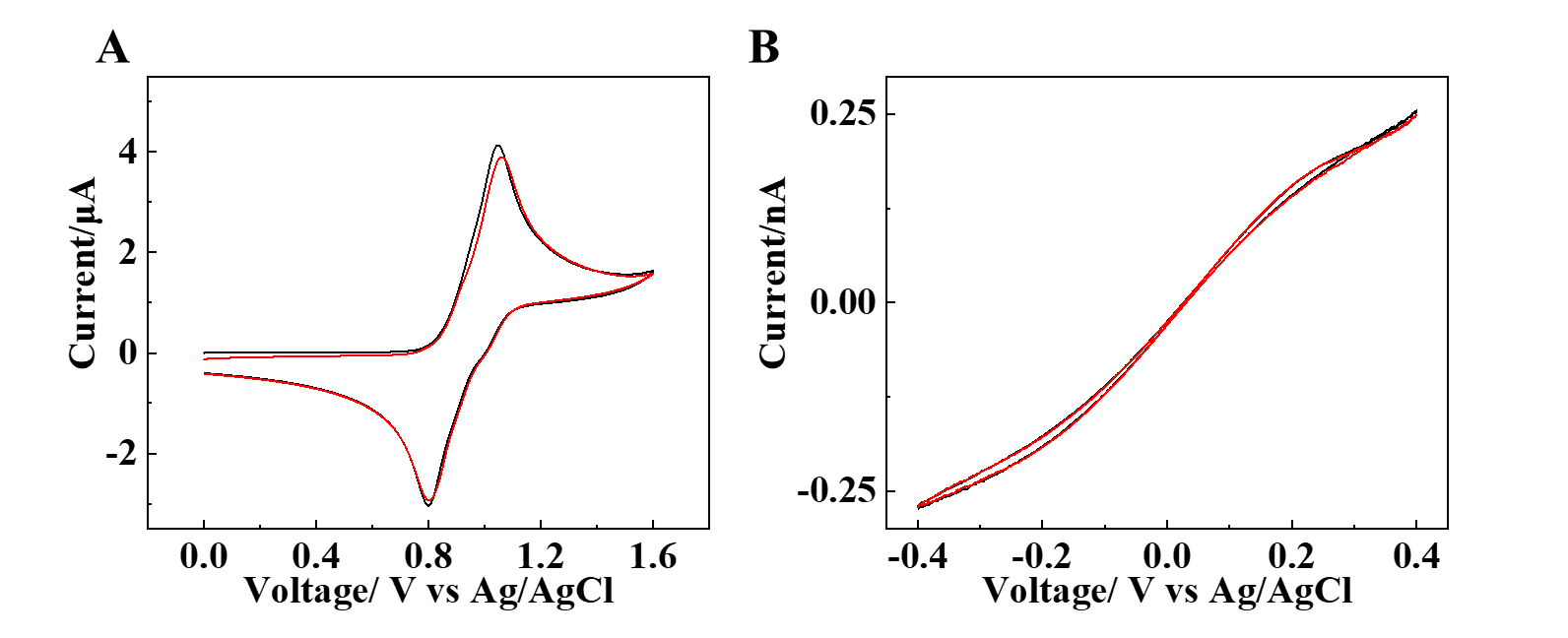
**

**Fig. S4.** The SEM images of (A) the orifice of the nanopipette and (B) the orifice with the solid-electrolyte nanoball.


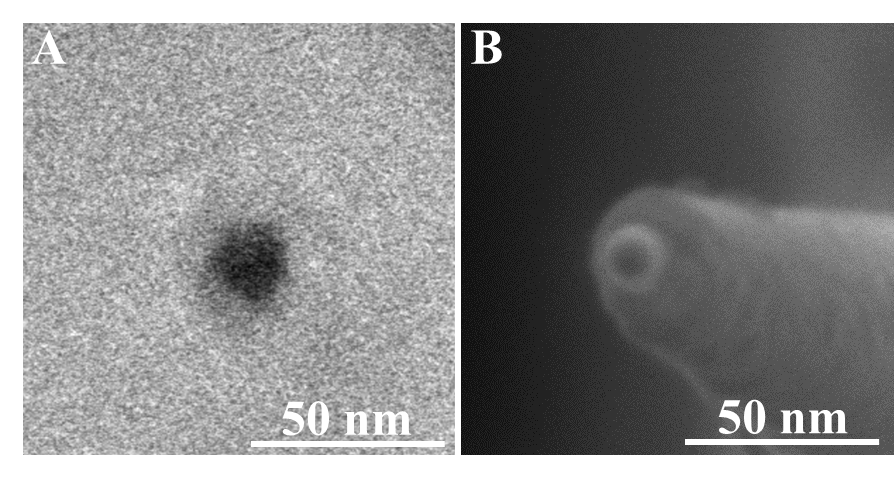


**Fig. S5.** The SEM image of the footprints after the nanoball is contact with Au coated ITO surface. A voltage of 1.4 V is applied at the Au/ITO electrode to complete the corrosion.

**Fig. S6.** Fluorescence and SECCM imaging of single molecules. (A) The schematic structure of Cy5 and Ru(bpy)_3_^2+^ co-tagged antibody–antigen immune complex at glutaraldehyde modified ITO surface; (B) single molecule fluorescence image of two molecules of immune complexes at ITO electrode using STED microscopy; (C) single molecule position achieved by 2D Gaussian fitting; (D) the photobleaching and photoblinking trace of single immune complex tagged with two Cy5 molecules.


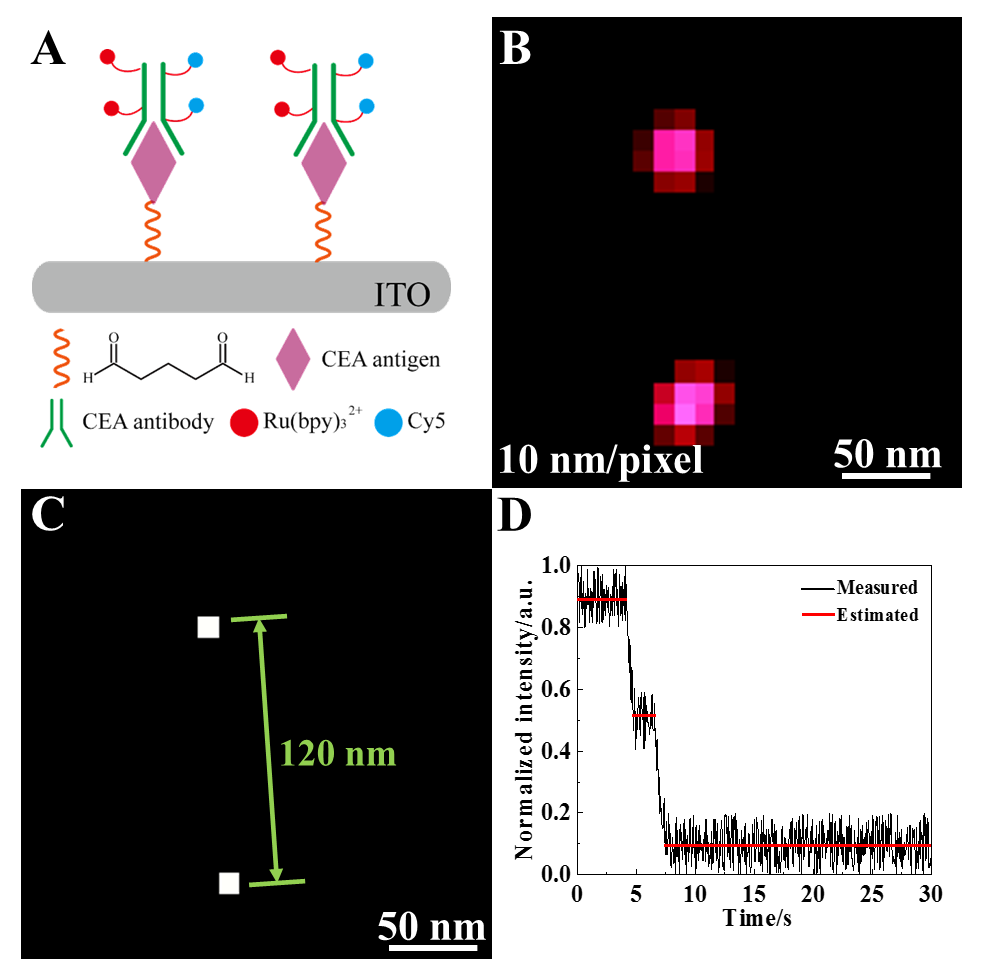


**Fig. S7.** The initial non-Faradic current upon the contact of the nanoball with Ru(bpy)_3_Cl_2_ film at ITO surface. A voltage of 1.23 V is applied at ITO surface. The red line is the fitting curve according to Randles equivalent circuit model. The fitting result is shown in Table S1.

**Fig. S8.** (A) Cyclic voltammetry of 0.5 mM Ru(bpy)_3_^2+^ in solid electrolyte with different scan rates; (B) the relation between ln(i_p_) and ($E_{p}-E^{\theta^{'}})$. The electrode area is 7 mm^2^.

Note: The relationship between peak current and potential can be described as:

$i_{p}=0.227FAC_{R}^{*}k^{0}exp\left[ -\alpha f\left( E_{p}-E^{\theta'} \right) \right]$ eq. S3

where i_p_ is the peak current, F is faradaic constant, A is working electrode area, $C_{R}^{*}$ is the initial reduction concentration, k^0^ is the reaction rate constant, α is the transfer coefficient, f is F/RT, E_p_ is the peak potential and $E^{\theta'}$ is the standard electrode potential. According to the result in the voltammetry, $E^{\theta'}$ can be achieved by $E^{\theta'}=\left( E_{O}+E_{R} \right)/2$, which is 1.0435 V. Once $E^{\theta'}$ is known, ln(i_p_) is in direct proportion to $E_{p}-E^{\theta'}$, which is shown in Figure S6B. The slope (–αf) is -9.74, and the intercept ($\ln\left( 0.227FAC_{R}^{*}k^{0} \right)$) is -12.46. Then k^0^ can be achieved to be 0.153 cm^-1^s^-1^. Suppose that the thickness of electric double layer is 1 nm, the time for the electron transfer from Ru(bpy)_3_^2+^ to Ru(bpy)_3_^3+^ in solid electrolyte is 0.64 μs.

**Fig. S9.** The typical electrochemical noise in the SECCM setup.

**Fig. S10.** The SECCM charge image of (A) the isolated single and (B) the dense distribution of immune-complexes at ITO electrode. The charge is integrated from the currents recorded in the time period of 20-30 ms.


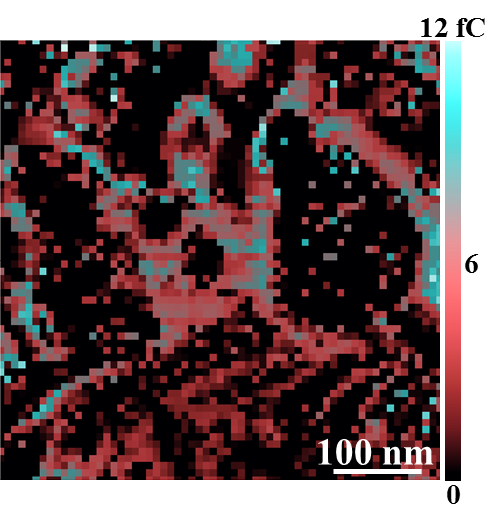


**Fig. S11.** SECCM morphology of the same single cell with scanning pixel of 128×128 using nanopipette with tip diameter of (A) 80 nm and (B) 20 nm; (C) height distribution along the red dashed line.

**
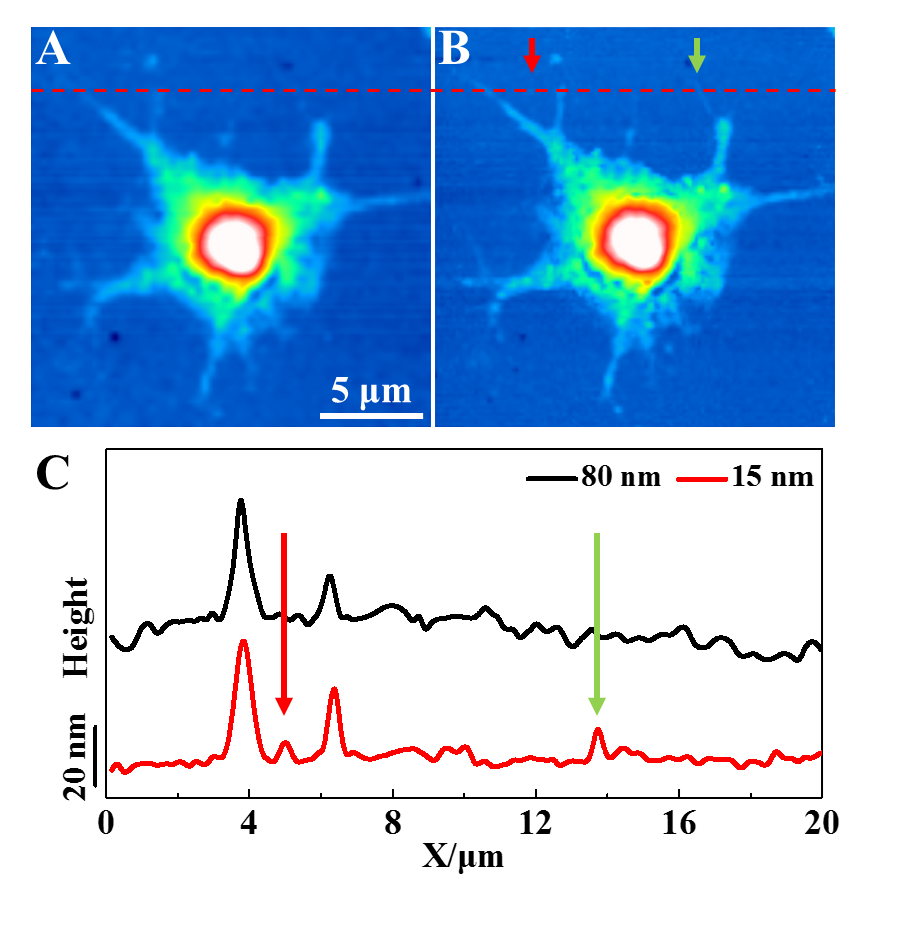
**

**Fig. S12.** The current traces of the nanoball contacted with Ru(bpy)_3_^2+^ tagged cell membrane at ITO electrode before (black curve) and after (red curve) the illumination. The shadow region presents the Faradic charges from the redox cycling of Ru(bpy)_3_^2+^ tag at the cellular membrane.


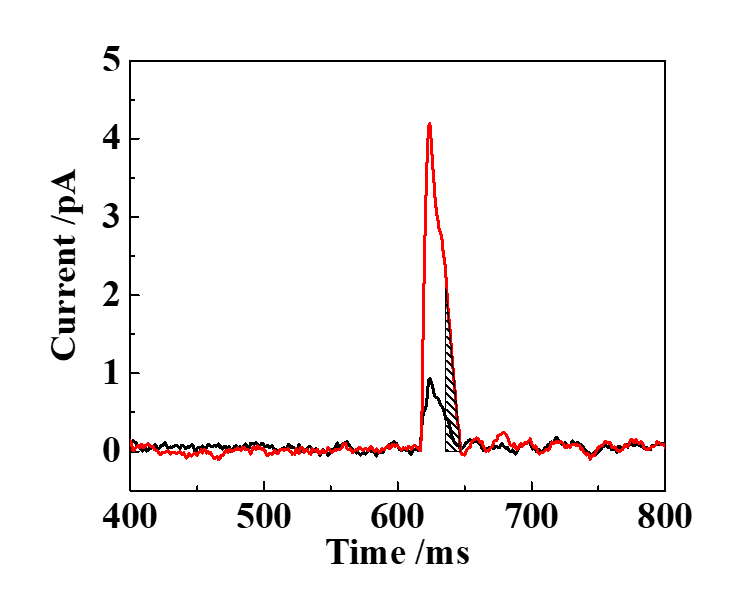


**Fig. S13.** CV results of cell surface using SECCM with scanning rate of 5 V/s.


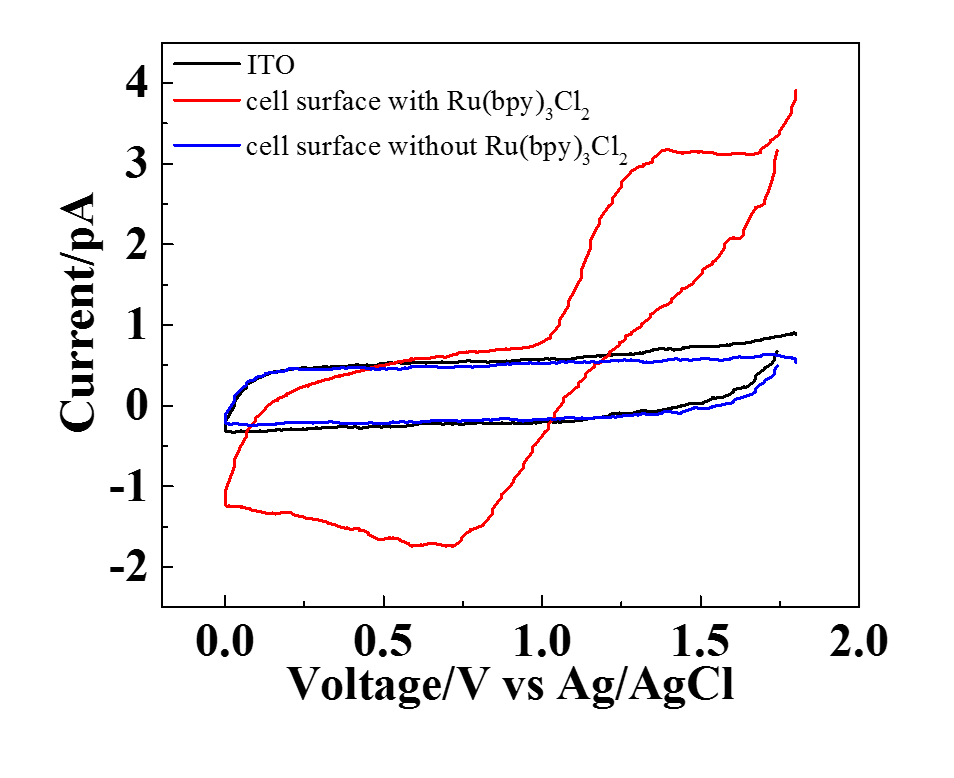


**Fig. S14.** The initial non-Faradic current upon the contact of the nanoball with (A) Ru(bpy)_3_^2+^ tag immune-complex and (B) Ru(bpy)_3_^2+^-Ab linked cell membrane at ITO surface. A voltage of 1.23 V is applied at ITO surface. The red line is the fitting curve according to Randles equivalent circuit model. The fitting results are shown in Table S1.


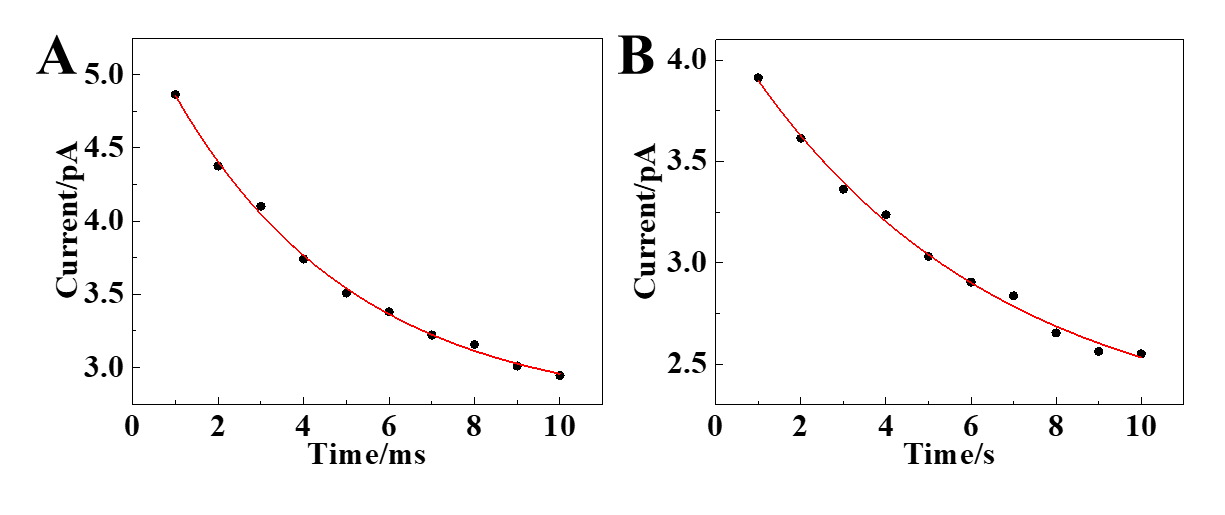


**Fig. S15.** The STED fluorescence and SECCM images of the cell margin tagged with Ru(bpy)_3_^2+^ and Cy5. (A) The bright field image and (B) fluorescence image of one MCF-7 cell; (C) the fluorescence image of cell margin as circled in the figure B; (D) the SECCM charge image of the same region as figure C. The scanning region is 640 × 640 nm (64 × 64 pixels) and the scanning interval is 10 nm.


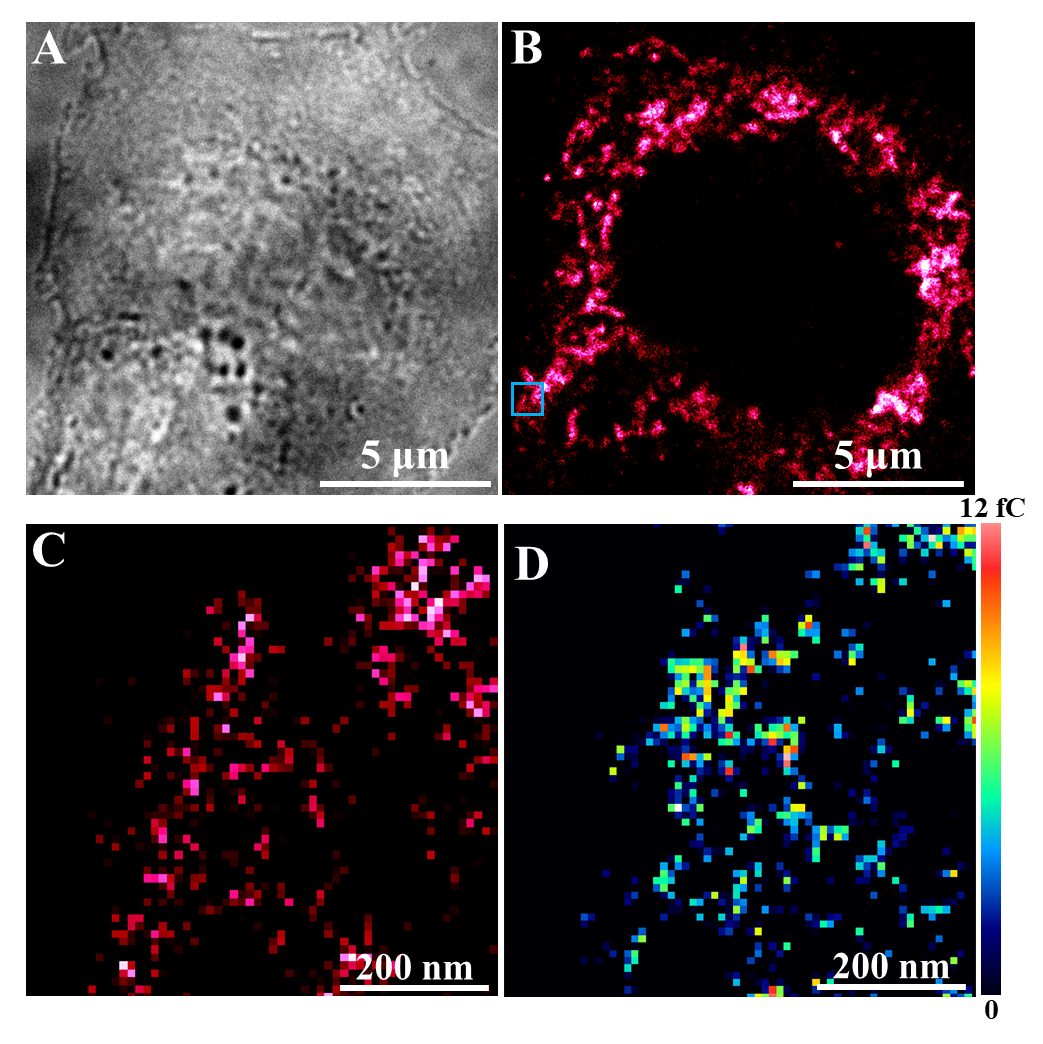


**Fig. S16.** (A) The SECCM topography image of single J774 cell; (B) the SECCM charge image that does not show any CEA antigen at the cellular membrane. The scanning region is 20 × 20 μm (128 ×128 pixels) and the scanning interval is 156 nm.

**Fig. S17.** The images from the replicate scanning of the whole cell tagged with Ru(bpy)_3_^2+^. (A, B) The SECCM topography images; (C, D) the SECCM charge images. The scanning region is 20 × 20 μm (128 ×128 pixels) and the scanning interval is 156 nm.

**Fig. S18.** The images from the replicate scanning of a small cell region. (A, B) The SECCM topography images; (C) the difference image (topography) from image A and B; (D, E) the SECCM charge images; (F) the difference image (charge) from image D and E. In the difference image (C: topography), only 97 out of 16384 points (0.59%) were observed. In the difference image (F: charge), only 142 out of 16384 points (0.87%) were observed. The scanning region is 1.28 × 1.28 μm (128 ×128 pixels) and the scanning interval is 10 nm.

**Table S1.** The stimulated result from the initial part of non-Faradic current when the nanoball is contacted with Ru(bpy)_3_Cl_2_ crystal, monolayer of Ru(bpy)_3_^2+^-Ab-Ag and Ru(bpy)_3_^2+^-Ab modified cell. R_s_ is the ionic resistance, R_f_ is the Faradaic resistance, C_dl_ is the double layer capacitance and τ is the time constant. The standard deviation is calculated from three independent measurements.

| Sample | R_s_/Ω | R_f_/Ω | C_dl_/F | τ/ms |
| --- | --- | --- | --- | --- |
| Ru(bpy)_3_Cl_2_ crystal | (5.3±0.41)×10^10^ | (2.67±0.26)×10^11^ | (1.48±0.08)×10^-13^ | 6.6±0.87 |
| Monolayer Ru(bpy)_3_^2+^-Ab-Ag | (1.36±0.12)×10^12^ | (5.49±0.61)×10^12^ | (3.89±0.26)×10^-15^ | 4.3±0.61 |
| Ru(bpy)_3_^2+^-Ab modified cell | (1.41±0.13)×10^12^ | (7.25±0.67)×10^12^ | (5.01±0.31)×10^-15^ | 5.9±0.52 |
